# Phytochemicals derived from *Nicotiana tabacum* L. plant contribute to pharmaceutical development

**DOI:** 10.3389/fphar.2024.1372456

**Published:** 2024-04-12

**Authors:** Wenji Zhang, Xiaoying Pan, Jiaqi Fu, Wenli Cheng, Hui Lin, Wenjuan Zhang, Zhenrui Huang

**Affiliations:** ^1^ Guangdong Provincial Engineering & Technology Research Center for Tobacco Breeding and Comprehensive Utilization, Key Laboratory of Crop Genetic Improvement of Guangdong Province, Crops Research Institute, Guangdong Academy of Agricultural Sciences, Guangzhou, China; ^2^ Department of Public Health and Preventive Medicine, School of Medicine, Jinan University, Guangzhou, China; ^3^ Department of Radiation Oncology, Guangdong Provincial People’s Hospital and Guangdong Academy of Medical Sciences, Guangzhou, China

**Keywords:** *Nicotiana*, neurodegeneration, inflammation, metabolic syndrome, nicotine, solaneso, cembranoid diterpenes, tobacco extracts

## Abstract

The *Nicotiana tabacum* L. plant, a medicinal resource, holds significant potential for benefiting human health, as evidenced by its use in Native American and ancient Chinese cultures. Modern medical and pharmaceutical studies have investigated that the abundant and distinctive function metabolites in tobacco including nicotine, solanesol, cembranoid diterpenes, essential oil, seed oil and other tobacco extracts, avoiding the toxic components of smoke, mainly have the anti-oxidation, anti-lipid production, pro-lipid oxidation, pro-insulin sensitivity, anti-inflammation, anti-apoptosis and antimicrobial activities. They showed potential pharmaceutical value mainly as supplements or substitutes for treating neurodegenerative diseases including Alzheimer’s and Parkinson’s disease, inflammatory diseases including colitis, arthritis, sepsis, multiple sclerosis, and myocarditis, and metabolic syndrome including Obesity and fatty liver. This review comprehensively presents the research status and the molecular mechanisms of tobacco and its metabolites basing on almost all the English and Chinese literature in recent 20 years in the field of medicine and pharmacology. This review serves as a foundation for future research on the medicinal potential of tobacco plants.

## 1 Introduction

Tobacco (*Nicotiana tabacum* L.), of the genus *Nicotiana* in the Solanaceae family, is an important economic crop and scientific research model plant. In recent years, beyond the cigarette industry, the innovative use of tobacco plants has inspired much research into organic fertilizers, biological pesticides, polymer biomaterials, food, feed, daily chemicals, and most notably, the medical field. Tobacco was first documented in human use at the end of the 15th century because of its medicinal value. Native Americans regard tobacco as a “holy herb” or “God’s remedy” used to treat a variety of diseases such as bronchitis, toothache, sore throat, pleuritis, jaundice, epilepsy, rhinitis, gastroenteritis, diarrhea, headache, otitis, whooping cough, syphilis, arthritis, dermatitis, colds, burns, abscess festering, and mosquito bites, and also applied for stopping bleeding, reducing fever, anti-fatigue, and whitening teeth (Sanchez-Ramos, 2020). In China, tobacco plants are also recorded as a characteristic medicinal resource. The Chinese Materia Medica documents the medicinal role of tobacco, including promoting “Qi” (vital energy) and relieving pain, eliminating dampness, detumescence, detoxicating and killing insects. It is mainly used to treat fullness with food stagnation, Qi stagnation with pain, arthralgia, carbuncle, furuncle, scabies, eczema, snakebite, sprain and contusion. The Compilation of National Chinese Herbal Medicine describes tobacco’s efficacy as warming, sweet, and toxic, with effects related to detumescence, detoxification, and insecticidal. It is primarily employed in treating furuncle, tinea capitis, psoriasis, alopecia, and snakebites, as well as diseases such as neck carbuncle, back carbuncle, wind phlegm, and crane knee, including bone tuberculosis and chronic suppurative knee arthritis.

The global smoking-banning campaign in the modern era also restricted investigation into the medicinal value of tobacco plants to a great extent. Only in the late 20th century, researchers turned their attention to diseases affecting brain and nerve development, especially neurodegenerative diseases with a high incidence among the elderly, such as Alzheimer’s disease and Parkinson’s disease. The medical value of tobacco was increasingly recognized and efforts to promote it are growing. As proposed by Professor Anne Charlton of Manchester University, we should put aside the prejudice caused by the adverse effects of smoking and, instead, systematically analyze tobacco plants to explore substances with medical treatment value (Charlton, 2004).

With advancements in pharmacology, the biological activity mechanisms of many medicinal plants and their metabolites have been clarified. In many cases, individual constituents are used for treatment after extraction and purification. About a quarter of the prescription drugs originate from plants (Goldstein and Thomas, 2004), such as the antimalarial drug artemisinin (Price, 2000), anticancer drug paclitaxel (Zhu and Chen, 2019), and cardiovascular drug ginkgolide (Tian et al., 2017). At present, the main medicinal constituents brought into focus in tobacco plants include the alkaloid nicotine, phenolic substances such as rutin, apigenin and quercetin, phenolic acids such as chlorogenic acid, ferulic acid and caffeic acid, organic acids such as citric acid and malic acid, terpenoids such as solanesol and cembranoid diterpenes, and tobacco essential oils such as α- and β-ionone. In recent years, with the popularization of metabolomics, other metabolites with pharmacological activity in tobacco plants, such as vitamin E, vitamin K1, scopolamine, mesmine, and ferruloyltyramine, have also been identified and isolated (Xia et al., 2014). In addition, it is worth mentioning that polysaccharides, polypeptides and sterols from plants also have important biological functions in the medical field. For example, plant polysaccharides can effectively regulate the balance of blood lipids and blood sugar in the human body, mediate immune activity, exert anti-cancer effects, and activate cell antioxidant activity (Jiao R. et al., 2016). Plant polypeptides exhibit antioxidant, antiviral and metabolic syndrome regulatory effects, especially blood pressure lowering effects (Chai et al., 2021). Phytosterols are effective in reducing cholesterol levels (Yuan, 2020). Besides, tobacco is rich in sugars (constituting 25%–50% of dry weight) and proteins (26%–29% of dry weight at the seedling stage and 12%–15% of dry weight at the mature stage), as well as phytosterols such as β-sitosterol, stigmasterol, and rape sterols. Therefore, tobacco will be an important plant resource for drug development in the future.

This review will focus on the status of the pharmacological research on the main and abundant functional substances of tobacco plants in recent 20 years. These include the alkaloid nicotine, terpenes (including solanesol and cembranoid diterpenes), as well as extracts from tobacco leaves and seed.

## 2 Nicotine

**FIGURE 1:**
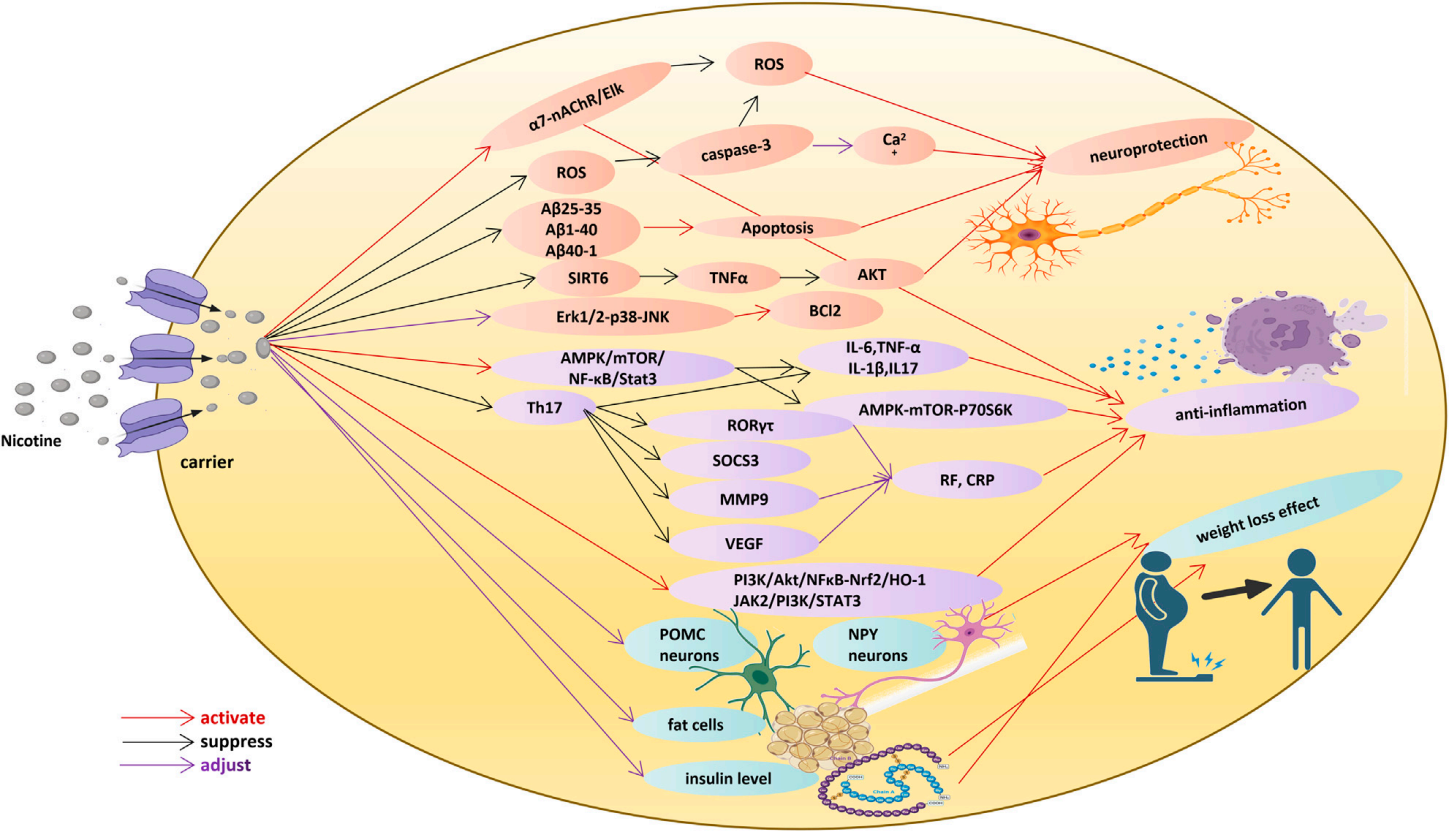
Nicotine function in neuroprotection, anti-inflammatory effect, and weight loss effect.

Nicotine (C_10_H_14_N_2_) is the main addictive substance in tobacco smoke, and belongs to the pyridine and pyrrole alkaloids, accounting for about 5% of the weight of tobacco and 90%–95% of the total alkaloids in tobacco. It mainly exists in tobacco, but is also found in a small amount in other solanaceous species. Alkaloids are nitrogen-containing compounds with remarkable anti-tumor, anti-microbial, anti-inflammatory and analgesic effects (Jiang et al., 2016). Nicotine, as an agonist of nicotinic acetylcholine receptor (nAChR), can act on the vagal pathway and its collateral branch and the anti-inflammatory pathway mediated by the nicotinic acetylcholine receptor, which is the main mechanism of nicotine’s pharmacological activity (Author Anonymous, 2014; Onor et al., 2017). The complex communication between the nervous system and immune system provides nicotine with a wide range of pharmacological activities and great potential for disease treatment. However, nicotine can be a double-edged sword. High-dose nicotine is toxic, while low-dose nicotine may have different positive effects, just like most clinical drugs. The negative effects of low-dose nicotine can be counteracted by other drugs (Barreto et al., 2014). Hereby, we introduce the application value of nicotine in the medical field, mainly focusing on neurodegenerative diseases, inflammation, and obesity metabolic disorders (Figure 1).

### 2.1 Positive effect of nicotine on neurodegenerative diseases

Neurodegenerative diseases are diseases of the nervous system caused by the selective degeneration and loss of neurons. Patients with these diseases will experience a decline in normal viability, resulting in problems with independent mobility, as well as memory and cognitive decline. Alzheimer’s disease and Parkinson’s disease are the two most common neurodegenerative diseases, which increases steadily with age and affect tens of millions of people worldwide (Erkkinen et al., 2018). Alzheimer’s is the most prevalent dementia disease worldwide, causing 60%–80% of all dementia cases. The pathogenesis of Alzheimer’s is complex. The neuronal damage and neurotoxicity caused by intracellular neurofibrillary tangles formed by β-amyloid protein (Aβ) deposition and abnormal phosphorylation of tau protein are the most important pathological features of Alzheimer’s (Alhowail, 2021). The pathogenesis of Parkinson’s mainly involves the loss or degeneration of dopamine neurons or dopamine-producing neurons in the substantia nigra of the midbrain (Barber et al., 2001). Nicotine administration can improve the cognitive impairment in Alzheimer’s, and the motor impairment and memory impairment in Parkinson’s, thus protecting nerve cells.

At the cellular level, nicotine activates the α7-nAChR/Elk signaling pathway to reduce the oxidative damage caused by hydrogen peroxide, improve damaged mitochondrial membrane potential, and inhibit the damage to hippocampal neurons induced by excessive accumulation of reactive oxygen species (ROS) (Dong et al., 2020). Nicotine weakens the apoptosis of primary hippocampal cells induced by Aβ_25-35_, Aβ_1-40_ or Aβ_40-1_ in Sprague-Dawley rats and reduces neurotoxicity by decreasing caspase-3 activity, clearing ROS and maintaining Ca^2+^ homeostasis (Liu and Zhao, 2004). In the Parkinson’s model of neuroblastoma SH-SY5Y cells induced by picoline ion (MPP^+^), nicotine pretreatment can activate α7 nAChR and further inhibit the cleavage of PARP-1 and caspase-3, resulting in a significant inhibition of cell death (Lu et al., 2017). In the Alzheimer’s model of SH-SY5Y cells, nicotine can inhibit the activity of caspase-3 of apoptotic protein and upregulate the expression of anti-apoptotic protein BCl2 through the Erk1/2-p38-JNK signaling pathway, thus protecting them from the neurotoxicity induced by Aβ_25-35_ (Xue et al., 2014). Nicotine can also promote the processing of AβPP non-amyloid protein mediated by α-secretase (ADAM10) and reduce the accumulation of neurotoxins via activating Rack1 protein-dependent PKC phosphorylation (He et al., 2020).

Nicotine has played a positive role in several animal models of Alzheimer’s disease. In Aβ_25-35_-induced Alzheimer’s mice, nicotine treatment increased the upregulation of anti-apoptotic protein BCl_2_ in the hippocampus of mice, while the Morris water maze navigation test proved that nicotine improved spatial working memory disorder (Xue et al., 2014). In a rat Alzheimer’s model induced by chloroindoleamine (CHL), Y maze and radial arm maze tests confirmed that nicotine significantly improved the spatial memory deficit, and restored the antioxidant capacity of superoxide dismutase in the rat hippocampus, showing potential properties for memory improvement (Hritcu et al., 2017). Through Y maze, new object recognition and radial arm maze assessment, nicotine metabolites cotinine and 6-hydroxy-L-nicotine, also showed cognitive improvement effects, positively regulating the expression of brain-derived neurotrophic factor (BDNF), neuronal specificity and postsynaptic memory consolidation related protein (arc) and proinflammatory factor interleukin one beta (IL-1β) in Alzheimer’s rats induced by Aβ, exhibiting higher affinity with α4β2 nAChR and α7 compared to nicotine. Hence, these metabolites may have greater potential as drug candidates for promoting cognition, antioxidation and anti-acetylcholinesterase (Boiangiu et al., 2020).

Nicotine also plays a neuroprotective role in several animal models with Parkinson’s disease. In 6-hydroxydopamine-induced Parkinson’s mouse. Nicotine can activate α7 nAChR receptor to play beneficial roles in motor deficiency, dopaminergic neuron loss, activation of astrocytes and microglia, and the decrease of dopamine in striatum via Wnt/β-Catenin signal transduction (Liu et al., 2017). In 1-Methyl-4-phenyl-1,2,3,6-tetrahydropyridine (MPTP) Parkinson’s mouse model, nicotine inhibits the activity of deacetylase SIRT6 or prevents the accumulation of SIRT6 molecules by mediating the degradation of proteasome, thus reducing the accumulation of inflammatory factor TNF-α, increasing the expression of phosphokinase AKT and signal transduction, and improving the survival rate of neurons (Nicholatos et al., 2018). Nicotine pretreatment has a neuroprotective effect on nigrostriatal injury in PD models, while the repair after injury has poor effects. This effect involves the mediation of various nAChR subtypes, including α4β2 nAChR, α6β2 nAChR and α7 nAChR, and may also involve changes in different downstream kinases, such as AKT, PI3K, and FGF2, thereby exerting a preprotective effect on injury (Quik et al., 2009). Therefore, we posit that the sustained release of a nicotine transdermal patch may avoid the desensitization readily caused by nicotine, and may be a healthcare product that can be promoted for preventing neural degeneration and improving life quality in the elderly in the future.

### 2.2 Anti-inflammatory effect of nicotine

The cholinergic anti-inflammatory pathway has been extensively studied in terms of its immune regulation function and protective effects on a variety of inflammation-related diseases, including ulcerative colitis, arthritis, sepsis, skin inflammation, multiple sclerosis, and myocarditis. The activation of the cholinergic anti-inflammatory pathway by nicotine verifies its extensive anti-inflammatory effects.

Ulcerative colitis is a non-transmural colitis disease, characterized by intermittent attack and remission, abdominal pain, diarrhea and rectal bleeding. Due to the complex pathogenesis of ulcerative colitis, available treatment protocols are limited and often fail to achieve satisfactory efficacy (Baumgart, 2009). According to epidemiological analysis, smokers have lower possibility of suffering from ulcerative colitis than non-smokers. The combination of nicotine patches for percutaneous treatment of cholinergic anti-inflammatory pathways with conventional treatment has shown better clinical effect than the single treatment (Kannichamy et al., 2020). However, the clinical sample size is small and the data is not sufficient. In animal models, subcutaneous injection, intraperitoneal injection and dietary nicotine can stimulate nAChR receptors, mainly the α7-subunit, but also the α3-, α5-, β2-and β4-subunits, inhibit proinflammatory cytokines such as IL-6, TNF-α, IL-1β, IL17 and IFN-γ produced by immune cells through the AMPK/mTOR/NF-κB/Stat3 inflammatory signaling pathway and AMPK-mTOR-P70S6K autophagy pathway, to alleviate ulcerative colitis induced by different inducers such as dextran sodium sulfate (Snoek et al., 2010; Lakhan and Kirchgessner, 2011; Gao et al., 2020). In addition, nicotine improves the integrity of intestinal wall cells (Costantini et al., 2012) by regulating cell tight junction proteins MAdCAM-1, occludin and ZO-1, thus accelerating wound healing. Due to the lack of specificity of nicotine in ulcerative colitis, nicotine clinical trials were halted. Hence, more preclinical research data on nicotine in ulcerative colitis is needed to determine the target and path of action so as to carry out multi-combination interventions.

Rheumatoid arthritis is characterized by chronic inflammation and synovial intima hyperplasia, which leads to cartilage deterioration and bone destruction, often lasting for several months (Disease et al., 2016). Under normal circumstances, there is a certain proportion of helper T cells 1 (Th1) and helper T cells 2 (Th2) in the body. The coordination between the two types of cells maintains the balance of the human immune system. When the Th1/Th2 ratio increases, indicating a shift towards Th1 immunity, the immune response produces a series of proinflammatory factors such as IL-1β, IL-6, IL-17, TNF-α, IFN-γ and IL-8, while the expression of anti-inflammatory factors such as IL-4, IL-5 and IL-10 declines, leading to the infiltration of inflammatory cells in synovial tissue (Gomes et al., 2018). Nicotine can induce the production of Th2 cells, reduce the level of GATA3, and block the progression of arthritis in mice after 33 days of collagen induction. In addition, nicotine can inhibit the immune response of Th17 cells, the cytokine IL-17 secreted by Th17 and its transcription factor RORγτ (Wu et al., 2014; Yang et al., 2014), and suppress cytokine signaling protein SOCS3 (Li et al., 2018), matrix metalloproteinase MMP9 and angiogenesis factor VEGF (Arshadi et al., 2020), to control rheumatoid factor (RF) and C-reactive protein (CRP), and reduce pannus formation and joint destruction in rheumatoid arthritis. The NF-κB and STAT-mediated intracellular anti-inflammatory signaling pathway and the vagus nerve play a key mediating role in the improvement of rheumatoid arthritis by nicotine (Li et al., 2010; Zhou et al., 2012).

Sepsis is a life-threatening organ dysfunction caused by the host’s maladjusted response to infection. If it is not identified or treated in a timely manner, it may lead to septic shock, multiple organ failure and even death (Singer et al., 2016). Through *in vivo* research, Wang et al. (Wang et al., 2004) first found that nicotine can reduce the mortality rate of mice with multimicrobial peritonitis from 84% to 51%, even when drug is administered to the mice after the occurrence of clinical disease. Nicotine could inhibit the activation of the NF-κB anti-inflammatory pathway through stimulating α7 nAChR and suppressing the secretion of high mobility group protein B (HMGB1), thereby inhibiting the production of proinflammatory cytokine plasminogen activator inhibitor-1 (PAI-1) as well as the microcirculation damage caused by sepsis. In septic mice infected by *Escherichia coli* or lipopolysaccharide injection, nicotine simultaneously reduced the proinflammatory factors of TNF-α, IL-6 and IL-1β in peritoneal lavage fluid and plasma and maintained the activities of serum enzymes AST and ALT of hepatic function so as to prevent liver failure and improve the survival rate by 40% (van Westerloo et al., 2005; Zhou et al., 2011). In acute sepsis induced by cecal ligation and perforation combined with lipopolysaccharide, the intraperitoneal injection of nicotine also actively reduces mortality in a short time through increasing intracellular calcium concentration by stimulating a7 nAChR and activating the anti-inflammatory signal pathways of PI3K/Akt/NFκB-Nrf2/HO-1 and JAK2/PI3K/STAT3. Nicotine has the capacity to reduce the oxidative damage of organs and tissues and it also prevents systemic inflammation and organ failure by regulating the oxidative stress system (Ozdemir-Kumral et al., 2017). In the treatment with nicotine, the splenic branch of the vagus nerve plays an indispensable role in the anti-inflammatory pathway (Huston et al., 2006; Rosas-Ballina et al., 2008). Microsomal prostaglandin E synthetase-1 gene (mPGES-1) and the PGE2 produced in the spleen are the key factors connecting the immune and nervous systems affected by nicotine (Revathikumar et al., 2018). Because sepsis, especially acute sepsis, only needs short-term treatment, concern that the long-term use of nicotine will adversely affect the nervous system is less significant here.

Skin inflammation is a general term that encompasses skin inflammatory diseases that are caused by various internal and external infections or non-infectious factors, and is not an independent disease. Due to its complex etiology and diverse clinical manifestations with repeated attacks, its clinical treatment is more difficult. The use of ultraviolet radiation exposure is a common and characteristic method to induce skin inflammation. An early intervention study in 1997 showed that a nicotine patch inhibited the skin’s inflammatory response to sodium dodecyl sulfate and ultraviolet radiation (Mills et al., 1997). By regulating α7 nAChR and SOCS3, oral administration of nicotine for 6 weeks significantly reduces the production of IL-1β in the skin of female rats exposed to ultraviolet light and skin redness and swelling were alleviated (Osborne-Hereford et al., 2008). Subcutaneous or intraperitoneal injection of nicotine in male Wistar rats increased the level of serum corticosterone and inhibited the increase of nitric oxide produced by polymorphonuclear leukocytes (mainly TNF-α), thus inhibiting the plasma exudation caused by passive skin reaction (Kubo et al., 2003; Kubo et al., 2004). Nicotine significantly reduced IL-8, IL-6 and VEGF levels in keratinocytes and endothelial cells co-cultured with the serum of Bechet patients (Kalayciyan et al., 2007), indicating that nicotine has therapeutic potential in reducing systemic vascular inflammation.

Multiple sclerosis is an autoimmune demyelinating disease characterized by inflammation of the central nervous system, demyelination and neurodegeneration, which is manifested by vertigo and weakness. The etiology of multiple sclerosis is still unknown and there is no method of complete cure. In a Swedish population-based study (7,883 cases, 9,437 controls), it was found that nicotine reduced the risk of multiple sclerosis in different subjects who received snuff, suggesting that nicotine plays an anti-inflammatory and immunomodulatory role in multiple sclerosis. Nicotine has a protective effect in the experimental autoimmune encephalomyelitis animal model of multiple sclerosis. Nicotine patch allows ependymal cells to proliferate in the inflammatory region by reducing the expression of nestin protein in neuroepithelial stem cells. It also has an obvious influence on the activity, activation and function of microglial cells, and can increase the number of mature anti-inflammatory M2 microglial cells (NG2^+^ and CC1^+^ subtype) so as to promote disease recovery (Gao et al., 2014; Gao et al., 2015). Nicotine also reduces the levels of pro-inflammatory factors IL-1, TNF-α and IFNγ and increases the levels of anti-inflammatory factor IL-10 in spleen cells, thereby improving the neurophysiological indexes. When nicotine is administered in combination with mesenchymal stem cells in rats, the efficacy is better than with the single treatment without nicotine (Khezri et al., 2018). It is worth noting that nAChRα7, α9 and β2 may play different roles under the action of nicotine. In clinical applications, it may be necessary to set specific inhibitors to make nAChR play a specific role, and the detailed mechanism needs to be further verified (Simard et al., 2013; Liu et al., 2017).

Myocarditis may cause symptoms from mild dyspnea or chest pain to cardiogenic shock and sudden death, and it can also be relieved without specific treatment. Myocarditis is usually caused by viral infection and, to date, there is no special treatment available (Cooper, 2009). In the myocarditis mouse model infected by coxsackie B3 virus, the injection of high concentration nicotine (1.2 mg/kg, i. p.) downregulated the expression levels of inflammatory factors IL-1β, IL-17A, TNF-α, IL-1 and IL-6 based on STAT3 activation, improved the damage to left ventricular function, and alleviated myocardial injury, increasing the survival rate by 35% (Cheng et al., 2014; Li-Sha et al., 2015; Li-Sha et al., 2016). Nicotine can also reduce the release of proinflammatory factors in the heart by regulating the proportion of immune cells Th1 and Th17 in the spleen (De-Pu et al., 2018). The nicotine-mediated reduction of coxsackie B3 virus replication and the anti-apoptosis effect on myocardial cells involve α7 and α3β4 nAChR, which further enhance cardiac function through upregulating the expression of survivin via the α3β4 nAChR/PI3K/Akt signaling pathway (Li et al., 2019). It is necessary to clarify the role of the vagus nerve’s integrity in this process.

Apart from the conditions mentioned above, nicotine plays an active role in many other inflammatory diseases, including pancreatitis, allergic inflammation, nasal eosinophilic inflammation, muscle inflammation, uveitis, anorexia/cachexia syndrome, and systemic lupus erythematosus. The effect of nicotine is more significant in inflammatory diseases with complex pathogenesis, which are difficult to cure and with recurrent attacks. Its advantage in multi targets may enhance its efficacy and improve individual survival rate, but it also suffers the problem of poor specificity. Therefore, the treatment of inflammatory diseases with nicotine depends on detailed studies on the acetylcholinergic anti-inflammatory pathway, the vagus nerve, the central nervous system regulation and the interaction with immune cells. Considering the addictive properties of nicotine, it is also critical to control the dosage. According to the approximate dosage range of nicotine tartrate (0.4–1.2 mg/kg per day) for intraperitoneal injection in mice, we calculated that the safe dosage range of nicotine salt for subcutaneous injection in humans is about 7.2–28.8 mg/kg per day in our previous report (Zhang et al., 2022). Currently, there are three concentrations of nicotine patches for smoking cessation treatment, namely, a 24-h patch with dosages of 21, 14, and 7 mg. Nicotine patches may be a potential choice for chronic recurrent inflammation (Zhang et al., 2022).

### 2.3 Weight loss effect of nicotine

Obesity has become a global public health problem. It is characterized by chronic low-grade inflammation, which can readily lead to nonalcoholic fatty liver disease, insulin resistance, cardiovascular disease, depression and other complications. Epidemiological studies have shown that there is a strong relationship between smoking and weight. In different ages, the weight of non-smokers is often higher than that of smokers. Quitting smoking may result in weight gain (Audrain-McGovern and Benowitz, 2011), which highlights the weight loss effect of nicotine from another perspective.

Nicotine has been shown to improve obesity mainly through four mechanisms. Firstly, nicotine regulates energy intake and reduces appetite by acting on hypothalamic neurons and the neuropeptide system related to food intake. Low-concentration nicotine (100–1,000 nM) has a regulatory effect on anorexic POMC neurons, NPY neurons, and secretin/orexin neurons in the hypothalamus, and can mediate neuropeptide Y, leptin, orexin and its receptors and uncoupling proteins, as well as the level and activity of neurotransmitters such as dopamine and monoamine, with the effect of controlling food intake and reducing obesity (Li et al., 2000; Huang et al., 2011). However, different nicotine intake methods will complicate its effectiveness.

Secondly, nicotine can reduce the size of fat cells and fat content by regulating fat decomposition and synthesis, and it can also raise the heat production of brown fat and transfer fat storage from adipose tissue to muscle utilization. In male Sprague-Dawley rats, after subcutaneous micro-pump injection of nicotine for 1 week, the body weight of rats was reduced by 37%, while fat pad reduction was 21%, and fat decomposition increased by 78%. Nicotine reduces the activity of lipoprotein lipase (LPL) in adipose tissue and increases the activity of LPL in muscle. This means that the intake of triglycerides in adipose tissue is reduced, the decomposition is increased, and the cells become smaller, while the consumption of triglycerides in myocardium and skeletal muscle is increased (Sztalryd et al., 1996). When CD-1^®^ IGS male mice were fed with feed containing nicotine, the mice exhibited body weight loss (19.7%), increased physical stamina and decreased respiratory exchange rate under the condition of no change in food intake, which also induced the decomposition of triglycerides in adipose tissue and weight loss. The most obvious change is that the weight of epididymal fat pad and the size of fat cells have significantly decreased while the heat production of brown fat has increased (Liu et al., 2018). The high expression of UCP1 protein and the increased binding of guanine nucleoside 5′-hydrogen phosphate in brown fat indicate that the metabolic rate of fat increases after nicotine injection (Arai et al., 2001), which confirms that nicotine acts directly on weight loss without the side effects of two-way regulation.

Thirdly, nicotine regulates insulin levels induced by obesity and glucose production (Seoane-Collazo et al., 2021). Nicotine fed with the diet increases the insulin level of mice 4.3-fold and improves the blood glucose level, without affecting insulin sensitivity (Liu et al., 2018). The plasma glucose level of male obese Zucker rats also decreased significantly after long-term oral administration of nicotine with drinking water, and the glycogen content, glycogen synthase activity and gluconeogenesis in the liver were significantly lower than those in the control group, indicating that nicotine can reduce insulin resistance in obese diabetic rats by reducing the release of glucose in the liver, thus reducing the level of blood sugar to some extent (Liu et al., 2003). Nicotine also significantly reduced the hyperglycemia level and incidence of type 1 diabetes in a mouse model, and prevented the insulin level from falling, which is closely related to nicotine’s effect on the expression profile from Th1 to Th2 cytokine and the amelioration of pancreatitis (Mabley et al., 2002).

Fourthly, nicotine alleviates steatohepatitis induced by adipose tissue inflammation and obesity. Nicotine can not only reduce body weight by stimulating triglyceride decomposition in adipose tissue and heat production in brown adipose tissue (Seoane-Collazo et al., 2014; Liu et al., 2018), but also significantly inhibits adipose tissue inflammation, and ameliorates the steatohepatitis of obese mice induced by genetic obesity and high-fat diet (Lakhan and Kirchgessner, 2011; Wang et al., 2011). In C57BL/6J (B6) obese mice induced by genetic obesity (db/db) and high-fat and high-sugar diet, nicotine inhibited the increase of F4/80 in obesity-induced inflammation and the levels of proinflammatory cytokines such as TNF-α, IL-6, IL-1β and iNOS in serum (Wang et al., 2011). Furthermore, in isolated Kupffer cells of the liver, nicotine could alleviate steatohepatitis by inhibiting the ERK/NF-κB/iκB signaling pathway by interacting with α7 nAChR (Zhou et al., 2015a; Zhou et al., 2015b; Chen et al., 2016; Li et al., 2016). As for Sprague-Dawley obese rats fed a high-fat diet, nicotine ameliorated liver damage and reduced lipid inflammatory indicators in the liver such as PPARγ, TNF-α and IL-6 by reducing endoplasmic reticulum stress (Seoane-Collazo et al., 2014). The regulatory effect of α7 nAChR on the action of immune factors MCP-1 and KC has been confirmed in stearic acid (C18:0) or TNF-α-induced 3T3-L1 adipocytes (Jiao et al., 2016) and α7 nAChR^−/−^ deficient mice (Wang et al., 2011). Nicotine osmotic pump was effective in alleviating steatohepatitis in Wistar rats induced by L-amino acid. Apart from the proinflammatory factors of TNF-α, IL-6, IL-1β, nicotine inhibits hepatocyte apoptosis by regulating apoptosis proteins Bax and caspase-3. In addition, the acetylcholine anti-inflammatory pathway involved in the hepatic branch of the vagus nerve is one of the essential links for nicotine to alleviate nonalcoholic steatohepatitis (Kanamori et al., 2017).

## 3 Solanesol

**FIGURE 2:**
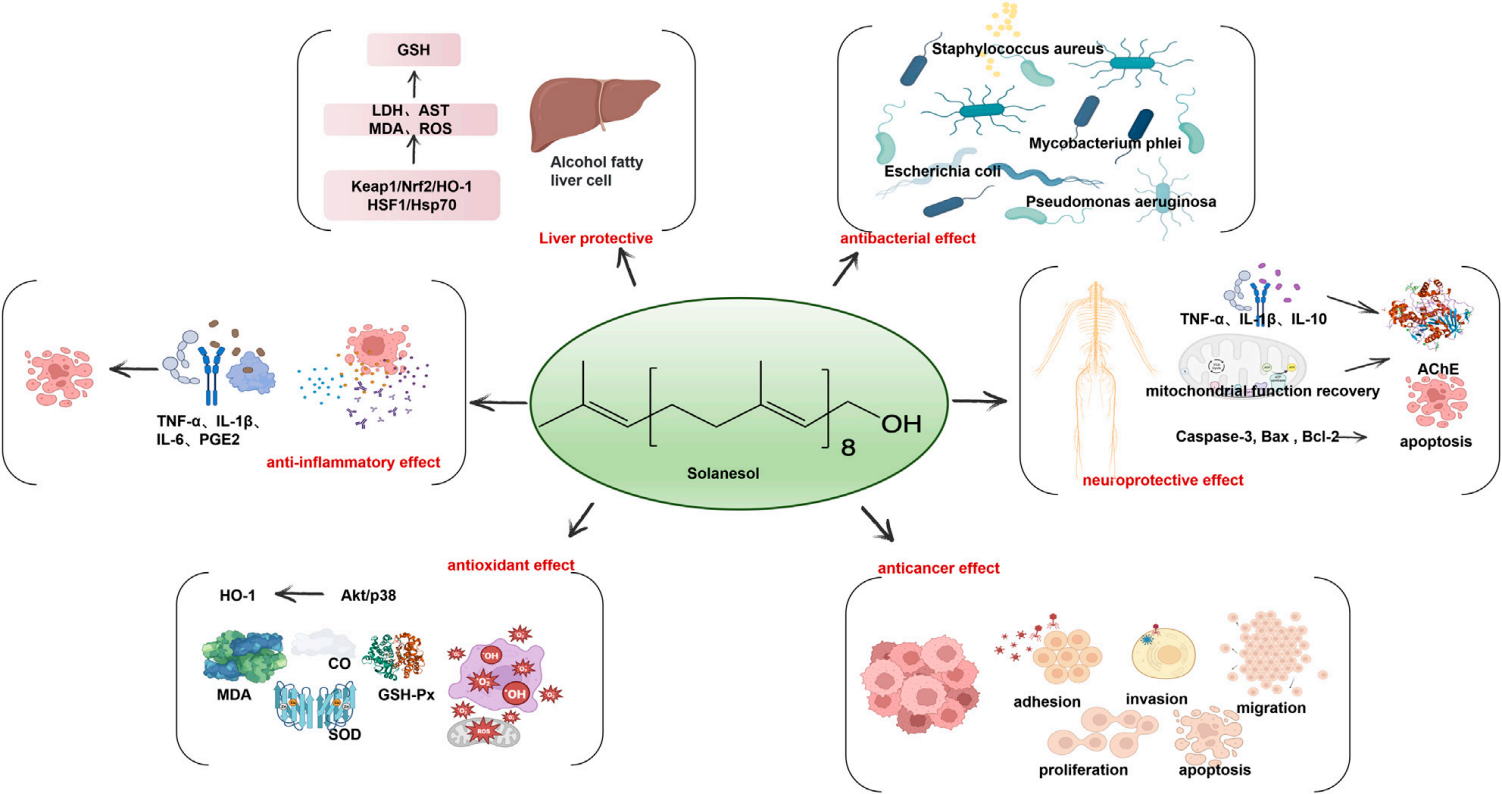
Solanesol has a wide range of biological activity including liver protection, anti-inflammatory effect, antioxidant effect, anticancer effect, neuroprotective effect and antibacterial effect.

Solanesol (C_45_H_74_O) is a long-chain triple sesquiterpenoid primary alcohol composed of nine isoprene units. Its special all-trans chain structure has lipid antioxidant activities and strong free radical absorption abilities. Additionally, solanesol also has antibacterial, anti-inflammatory and anti-ulcer biological activities (Figure 2). It is the raw material for the preparation of ubiquinone drugs such as vitamin K2, coenzyme Q10 (CoQ10) and N-solanesyl-N, N′-bis(3,4-dimethoxybenzyl) ethylenediamine, an anticancer agent synergiser. Tobacco is the most abundant source of plant solanesol, accounting for about 1%–4% of the dry weight. Due to the complex synthesis of solanesol, it is currently mostly extracted from tobacco (Yan et al., 2019a).

### 3.1 Antioxidant and anti-inflammatory activities of solanesol

Solanesol has many non-conjugated double bonds in its structure, which possess strong free radical absorption ability and antioxidant activity. Free radicals are products of respiration and metabolism in the human body. Under normal circumstances, the generation and elimination of free radicals are in a state of dynamic balance. Free radicals play an indispensable role in the normal growth of cells, energy metabolism and internal environment stability. However, when excessive free radicals are generated in the human body, they cause oxidative stress damage to cells, tissues and organs, induce various diseases, and accelerate the aging of the body. Under pathological conditions, excessive free radicals further aggravate diseases through oxidative stress. More than 95% of free radicals in the human body are oxygen free radicals including O_2_^• -^, HO_2_^•^, LO^•^, LOO^•^, NO^•^ and HO^•^. Of these, the hydroxyl free radical (HO^•^) displays the strongest activity (Droge, 2002).

*In vitro* studies show that in the pyrogallol autoxidation system and Fenton reaction system, solanesol scavenges O_2_^•-^and HO^•^, with an equivalent scavenging effect to vitamin C (Vc) and antioxidant Trolox (Ma et al., 2011; Bai et al., 2014). The Vc/Fe^2+^ excitation system and microsome model of lipid peroxidation *in vitro* have shown that lipid peroxidation can be inhibited by solanesol (IC_50_ = 2.5 mM) (Ma et al., 2011). In lipopolysaccharide (LPS)-induced inflammatory injury in RAW264.7 macrophages of mice, solanesol enhances the transcriptional level of Nrf2 by activating Akt and p38 signaling pathways, which further upregulates the expression level and the activity of heme oxygenase-1 (HO-1), and improves the antioxidant capacity of cells. It can also trigger the anti-inflammatory pathway and reduce the expression of inflammatory factors TNF-α, IL-6 and IL-1β, thus inducing autophagy and alleviating cell inflammation (Yao et al., 2017).

In *vivo* antioxidant models, solanesol has a protective role in aging mice and inflammatory mice based on its antioxidant effect. In the D-galactose-induced aging mouse model, solanesol increases the activity of superoxide dismutase (SOD) in brain tissue and serum, and decreases the content of malondialdehyde (MDA) and nitric oxide in serum. This shows that solanesol protects the brain, which is particularly vulnerable to oxygen free radicals, and has a certain ability to remove nitrogen free radicals (Ma et al., 2011). In the periodontitis rat model established by ligation of bilateral maxillary second M by silk thread combined with viscous high-sugar diet for 4 weeks, solanesol shows no obvious effect on reducing alveolar bone loss or inhibiting the activation of osteoclasts, but it significantly increases the activities of SOD and glutathione peroxidase (GSH-Px) in plasma and reduces the content of MDA. Furthermore, it significantly lowers the expression level of inflammatory factors TNF-α, IL- 1β and PGE2 in plasma (Zhang, 2016).

In addition, the fat solubility of solanesol is more conducive to its exerting free radical scavenging ability in the lipid part of the skin. Excessive production of free radicals in the skin can cause pigmentation, resulting in reduced skin elasticity, skin aging and even cancer. Under the external stimulator of ultraviolet light, solanesol exhibits an inhibitory rate of more than 90%. By applying solanesol to fresh pig skin, solanesol alleviates the degree of skin atrophy and browning, and has whitening and anti-aging effects by inhibiting the activity of tyrosine kinase that induces skin melanin production (Bai et al., 2014).

### 3.2 Liver protective activity of solanesol

The induction of HO-1 by solanesol in different cells may be a universal mechanism. In human liver LO2 cells with ethanol-induced oxidative damage, solanesol alleviated hepatotoxicity by activating Keap1/Nrf2/HO-1 and HSF1/Hsp70 signaling pathways, significantly inhibiting the activities of lactate dehydrogenase (LDH) and AST in injured hepatocytes, and blocking ethanol-induced upregulation of MDA and ROS levels and downregulation of glutathione (GSH) levels. Solanesol could further inhibit cell apoptosis by inhibiting the morphological damage of the nucleus and the mature shear of apoptotic protein caspase-3 and PARP, suggesting that solanesol may have liver protective effects in alcoholic fatty liver (Yao et al., 2015). However, it is worth noting that LO2 cells are contaminated with Hela tumor cells, and the results obtained as an induction model are questionable (Shao and Chen, 2023).

### 3.3 Neuroprotective effect of solanesol

Solanesol has a neuroprotective role by regulating neuroinflammatory factors TNF-α, IL-1β and IL-10, and the activity of mitochondrial complexes such as NADPH dehydrogenase, succinate dehydrogenase (SDH), ATP, and CoQ10. It also inhibits brain oxidative damage through enhancing SOD and GSH and reducing MDA and nitrite, restoring the levels of neurotransmitters acetylcholine and acetylcholinesterase (AChE), and regulating brain diseases by mediating biochemical indicators *in vivo* such as dopamine (Mehan et al., 2018; Sharma et al., 2019; Rajdev et al., 2020). In the combined rat model of intracerebral and ventricular hemorrhage, solanesol significantly ameliorated behavior and movement disorders, oxidative damage and neuroinflammation, and restored the activity of mitochondrial complexes (I, II, and V) in hemorrhagic rats after 35 days of treatment, indicating that solanesol has multiple-target activities in various cellular and molecular cascade reactions. When solanesol is combined with standard drug treatments (donepezil, memantine, celecoxib, and pregabalin) to treat symptoms after intracerebral hemorrhage in rats, it has a synergistic effect (Rajdev et al., 2020). In an experimental model of autism induced by propionic acid injection into the lateral ventricle, solanesol restored the complex enzyme levels in the mitochondrial electron transport chain. Long-term treatment can benefit the recovery of memory and the decrease in the level of neuroproinflammatory factors, and restore neurotransmitter levels, slow down oxidative stress, improve dopamine levels, enhance exercise ability and exhibit antidepressant activity (Sharma et al., 2019). In the experimental model of Huntington’s disease induced by 3-nitropropionic acid, low-concentration solanesol improved the function of mitochondria and acted on the striatum, cortex and hippocampus of the brain to promote the recovery of brain energy, enhance the level of acetylcholine, improve memory and motor ability, strengthen the antioxidant defense system of the brain, and alleviate Huntington’s disease (Mehan et al., 2018). A recent study also shows that solanesol can ameliorate ouabain-induced bipolar disorder by activating the SIRT-1 signaling pathway (Rajkhowa et al., 2022). Data demonstrate that solanesol can increase the level of SIRT-1 in cerebrospinal fluid, plasma and brain homogenates samples and regulate apoptosis markers (Caspase-3, Bax and Bcl-2), mitochondrial complexes I, II, IV, V and CoQ10. It can also reduce inflammatory cytokines TNF-α and IL-1β to simultaneously restore the level of neurotransmitters, including serotonin, dopamine, glutamate and acetylcholine, and reduce the markers of oxidative stress, thereby limiting the severity of this chronic mental disease. Hence, solanesol has the effect of alleviating neuroinflammation and improving neuronal memory and motor dysfunction. It can also change neurochemical reactions and enhance the cerebral antioxidant defense in a neuroprotective role.

### 3.4 Antibacterial activity of solanesol

Based on *in vitro* bacteriostasis experiments using the agar diffusion method and double dilution method, solanesol exhibits significant inhibitory effects on *E. coli*, *Mycobacterium phlei*, *Pseudomonas aeruginosa* and *Staphylococcus aureus*. However, it has a poor inhibitory effect on *Bacillus circulans* and *Bacillus subtilis* (Chen et al., 2007).

### 3.5 Pharmacological activities of solanesol derivatives

#### 3.5.1 Anticancer effects of solanesol derivatives

Many natural products enjoy a reputation as promising anticancer agents. A number of compounds based on dietary isoprenoids have attracted attention due to their chemopreventive and anti-proliferative properties. Some little-known isoprenoid derivatives have gradually been applied in mainstream chemotherapy (Newman and Cragg, 2016). As an isoprenoid compound, solanesol was recognized by molecular dynamics analysis to be an inhibitor of focal adhesion kinase (FAK) protein to block its binding to ATP. The FAK protein participates in cell adhesion, invasion, migration, proliferation and apoptosis and is involved in tumor occurrence, development and prognosis (Daneial et al., 2017).

A derivative of solanesol, *N, N′*-bis(3,4-dimethoxybenzyl)-*N*-solanesyl ethylenediamine has been screened with multiple tumor cell lines. It is not only toxic to tumor cells, but can also mediate the multidrug resistance protein p-gp glycoprotein to restore sensitivity of tumor cell lines to the anticancer drugs vinblastine and doxorubicin (Sidorova et al., 2002). *Trans*-*N, N′*-bis(3,4-dimethoxybenzyl)-*N*-solanyl 1,2-diaminocyclohexane (N-5228) can also completely reverse the effect of human bladder cancer drug-resistant cell line KK47/TX30 on paclitaxel by mediating p-gp (Enokida et al., 2002).

Solanesol can also be utilized to modify traditional anti-cancer drugs to enhance anti-tumor activity, improve the bioavailability and precision targeting ability of insoluble drugs, and reduce the toxicity of anti-cancer drugs to normal cells. The 12 synthesized derivatives of the dibasic acid solanesol alkyl 5-fluorouracil diester inhibited the proliferation of human lung cancer cell A549 and colon cancer cell HCT1169 to varying degrees, and solanesol itself presents certain inhibitory effects on tumor cell proliferation (Xiao et al., 2012). Recently, the research team of Henan University reported amphiphilic solanesol derivatives of methyl poly (ethylene glycol) (mPEG) with solanesyl thiosalicylate (mPEG-STS), solanesyl thiosalicylate hydrazone (mPEG-HZ-STS), solanesyl dithiodipropionate (SPDP), solanesyl succinate (SPGS), and the solanesyl thiosalicylic acid (STS) polymer (HA-STS) designed with hyaluronic acid (HA) as the basic skeleton, which exhibit inhibitory effects on breast cancer and liver cancer cells. The micelles loaded with doxorubicin, which were more active than those loaded with blank micelles, displayed significant antihepatoma activity in H22 tumor-bearing mice. Among them, SPDP, mPEG-HZ-STS and HA-STS loaded with doxorubicin present the highest effects (Qin et al., 2017; Xiong et al., 2019; Yu, 2019).

#### 3.5.2 Solanesol as raw material for drug synthesis

Solanesol is the key raw material for the synthesis of ubiquinone drugs including CoQ10 and vitamin K2. CoQ10 plays a pivotal role in mitochondrial oxidative phosphorylation and acts as a lipid-soluble antioxidant. It is involved in fatty acid, pyrimidine and lysosomal metabolism and directly mediates the expression of many genes, including those involved in inflammation. CoQ10 can be used to treat hypertension, and neurodegenerative, cardiovascular and other diseases, and is applied as a dietary supplement for patients with type 2 diabetes, heart failure, chronic kidney disease and liver disease (Hargreaves et al., 2020). Vitamin K2 is a lipid-soluble vitamin implicated in bone metabolism, which plays an active role in the prevention and treatment of osteoporosis. In addition, it can promote blood coagulation and prevent vascular calcification (Schwalfenberg, 2017).

## 4 Tobacco cembranoid diterpenes

**FIGURE 3:**
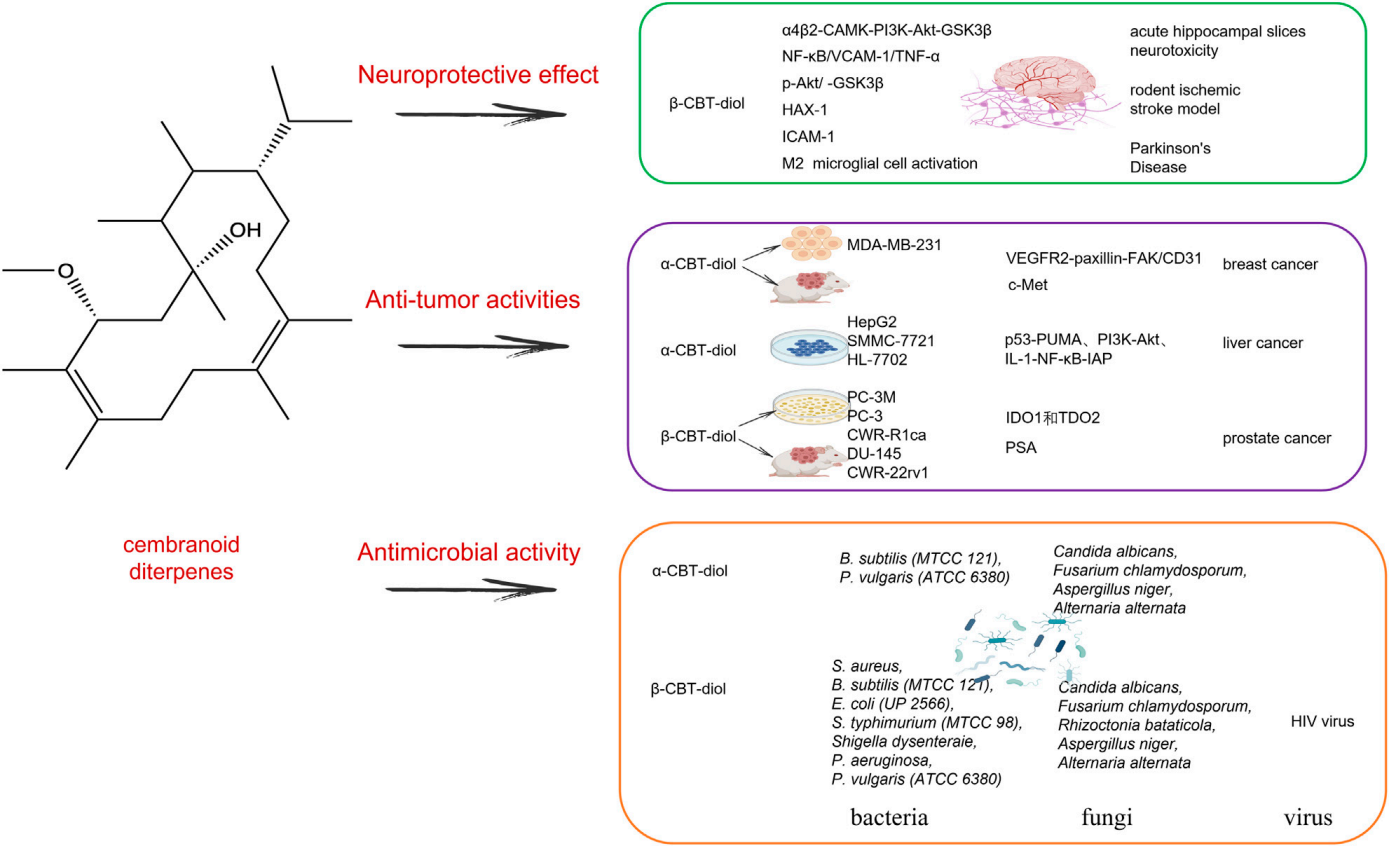
The mechanism effect of cembranoid diterpenes mainly including α-CBT-diol and β-CBT-diol in neuroprotective effect, anti-tumor activities and antimicrobial activities.

Cembranoids are natural diterpenes possessing 14-membered macrocyclic rings substituted by an isopropyl residue at C-1 and three symmetrically disposed methyl groups at positions C-4, C-8, and C-12 (El Sayed and Sylvester, 2007). The earliest discovered natural cembranoid was the (+)-cembrene from pine oleoresin, later it was found that the leaf and flower cuticular wax of most Nicotiana species afforded high amounts of the cembranoids, and they are the main source of tobacco aroma substances (Mudhish et al., 2022). Most useful bioactive cembranoids are intentionally broken down during commercial tobacco fermentation to produce some unique flavor, in fact, these natural cembranoids have shown several biological potentials, including antitumor, neuroprotective and antibacterial activity. Here, we mainly reviewed two major valuable substances that have been continuously updated (1S,2E,4R,6R,7E,11E)-2,7,11-cembratriene-4,6-diol (β-CBT-diol) and (1S,2E,4S,6R,7E,11E)-2,7,11-cembratriene-4,6-diol (α-CBT-diol) (Figure 3).

### 4.1 Anti-tumor activities

α-CBT-diol and β-CBT-diol mainly played a role in inhibiting the growth, invasion and recurrence of tumor cells in breast cancer, liver cancer and prostate cancer. α-CBT-diol could control invasive and metastatic breast malignancies mainly through angiogenesis inhibition (Ebrahim et al., 2016; Hailat et al., 2017).

In one breast cancer research, α-CBT-diol showed a strong affinity with VEGFR2 at its ATP binding pocket *in silico*. Both *in vitro* in TNBC MDA-MB-231 cells and *in vivo* in the implanted orthotopic MDA-MB-231 cells xenograft in athymic nude mice, α-CBT-diol significantly downregulated the activated VEGFR2-paxillin-FAK axis. Also, *in vivo,* it showed statistically significant reduction in tumor size, CD31 value, and markedly more active than *in vitro* because of the longer half-life, stability and the more active metabolites with allylic hydroxylation at C-19 and/or C-20 methyl carbons. Furthermore, the formation of blood vessels in matrigel injected in mice showed α-CBT-diol significantly reduced hemoglobin concentration (about 80% reduction) values when compared with vehicle-treated control, indicating α-CBT-diol is an effective angiogenesis inhibitor and would be useful to control of VEGF-dependent breast malignancies in the future (Hailat et al., 2017). The same group also found that α-CBT-diol has the antiproliferative, anti-migratory and anti-invasive effects against multiple breast cancer cell lines which the c-Met overexpressing MDA-MB-231 breast cancer cells are the most sensitive, indicating that α-CBT-diol may be a novel c-Met inhibitors for the c-Met dependent malignancies therapy (Ebrahim et al., 2016).

In liver cancer, α-CBT-diol inhibited the proliferation of HepG2, SMMC-7721 and HL-7702 tumor cells, reduced the formation of carcinoma cell clones, disrupted the cell cycle by significantly increasing S phase, induced cell apoptosis by regulating p53-PUMA, PI3K-Akt, and IL-1-NF-κB-IAP pathways (Yuan et al., 2019). But the genes involved in the pathways in the study were identified using Illumina sequencing in HepG2 cells, the differentially expressed genes need further verification *in vivo*.

In prostate cancer, β-CBT-diol showed its advantage in several pathways for the treatment of prostate cancer, especially for its higher recurrence. Earlier in the 2008, in PC-3M prostate cancer cells, β-CBT-diol has been proven to inhibit cell invasion in Matrigel assay, through reducing transepithelial resistance, enhancing paracellular permeability, producing a tighter intercellular barrier and enhancing the cell-cell adhesion (El Sayed et al., 2008). A recent study in 2022 further verified anti-prostate cancer activity of β-CBT-diol in multiple cell lines, tumor model, and biomarkers analysis (Mudhish et al., 2022). β-CBT-diol inhibited the viability of five prostate cancer cell lines PC-3M、PC-3、CWR-R1ca、DU-145 and CWR-22rv1, reduced their migration and colony formation. β-CBT-diol reduced heme-containing enzymes IDO1 and TDO2 expression in the PC-3M cell line which has a higher metastatic potential compared to other prostate cancer cell lines, indicating its important roles in cancer cell motility and immune escape. *In vivo*, β-CBT-diol inhibited PC-3M cells locoregional recurrences after primary tumor surgical excision and tumor distant recurrence, and reduced the levels of plasma kynurenine and the recurrence protein marker PSA in mice (Mudhish et al., 2022). β-CBT-diol, as a small-molecule natural product suitable for the control of hormone-independent prostate cancer recurrence, may have a broader application scope.

### 4.2 Neuroprotective effect

In the past 20 years from the year 2001–2022, the study on the neuroprotective effects of cembranoids has not stopped (Ferchmin et al., 2001; Ferchmin et al., 2005; Ferchmin et al., 2014; Martins et al., 2015; Hu et al., 2017; Fu et al., 2022). Since tobacco cembranoids were firstly discovered that they can be as the noncompetitive inhibitors to block the sensitization of nicotine by blocking agonist-induced ion current mediated by three distinct human AChR subtypes α4β2, α3β4, α1β1γδ-AChR (Ferchmin et al., 2001), β-CBT-diol not α-CBT-diol was later found to play a major role in neuroprotection by protecting acute hippocampal slices against neurotoxicity induced by N-methyl-D-aspartate and against the toxic organophosphorus compounds paraoxon and diisopropylfluorophosphate (Ferchmin et al., 2005; Eterović et al., 2013; Ferchmin et al., 2014). β-CBT-diol protected hippocampal sections from excitatory neurotoxicity induced by N-methyl-D-aspartate by modulating α4β2-CAMK-PI3K-Akt-GSK3β pathway, different from the cell-signaling pathways α4β2-PI3K-Erk-PKC underlying the neuroprotection provided by nicotine (Ferchmin et al., 2005). β-CBT-diol also ameliorated the damage caused by diisopropylfluorophosphate in the hippocampal area CA1 in rats, as a concrete manifestation of the decreased number of dead neurons by half when injected 1 h before or 24 h after diisopropylfluorophosphate and the significantly decreased number of activated astrocytes, by Fluoro-Jade B and amino cupric silver staining and nestin expression measurement (Ferchmin et al., 2014).

β-CBT-diol also decreased brain damage in rodent ischemic stroke models (Martins et al., 2015). β-CBT-diol treatment significantly reduced infarction size by more than half in brain ischemic stroke in mouse 10 min before permanent middle cerebral artery occlusion and rats after at 1 h after reperfusion, indicating β-CBT-diol may play a huge role in treating and preventing ischemic stroke. This has something to do with the β-CBT-diol, which can cross the blood-brain barrier and accumulate in the brain. It was further found that β-CBT-diol inhibited the expression of ICAM-1 in brain-derived endothelial cells and restored the phosphorylation of Akt that was stimulated by oxygen-glucose deprivation, thereby protecting neurons from inflammatory injury and death in ischemic stroke.

β-CBT-diol improved Parkinson’s disease both *in vitro* neuro-2a cell models and *in vivo* rat models induced by 6-Hydroxydopamine challenge (Hu et al., 2017). β-CBT-diol treatment improved rat behavioral deficits by 4 weeks after injection of 6-Hydroxydopamine by decreasing the depletion of tyrosine hydroxylase in the striatum and substantia nigra, as indicated by comparing forelimb asymmetry scores and corner test scores. β-CBT-diol also protected the viability of neuro-2a cells by activating anti-apoptosis protein p-Akt and HAX-1, inhibiting pro-apoptosis protein caspase-3, and regulating the NF-κB/VCAM-1/TNF-α pathway to resist endothelial inflammation in murine brain-derived endothelial bEND5 cells. Thus, β-CBT-diol may be a promising therapeutic agent for Parkinson’s disease.

The most recent study in the year of 2022 reported that β-CBT-diol protected neuronal cells from oxygen-glucose deprivation by modulating microglial cell activation (Fu et al., 2022), further provided data support for the study of β-CBT-diol as a drug for the treatment of ischemic stroke and neurodegenerative diseases. It has been found that β-CBT-diol exerted anti-inflammatory effects on microglia by regulating the M1/M2 phenotype to protect neurons from ischemia damage or inflammation. In LPS and oxygen-glucose deprivation -induced inflammation models of microglia N9 cells, β-CBT-diol promoted a neuroprotective tilt of microglia activation by down-regulating levels of NF-κB/iNOS, a marker of M1 inflammatory response, and up-regulating levels of Arg-1 and IL-10, markers of M2 anti-inflammatory activation. At the same time, β-CBT-diol produced beneficial factors in N9 medium, which further had a protective effect on oxygen glucose deprivation -induced Neuro2a cell damage, when the conditioned medium of β-CBT-diol-treated N9 cells was added to Neuro2a cells and incubated for 24 h. This study only detected a few markers at the cellular level, and future neuroprotective studies on β-CBT-diol require more in-depth exploration of molecular mechanisms at *in vivo*, so as to make adequate preparation for clinical application.

### 4.3 Antimicrobial activity

β-CBT-diol is very promising and has a broader spectrum of antimicrobial activities than α-CBT-diol (Aqil et al., 2011). β-CBT-diol showed good activity against the bacteria including *S. aureus, B. subtilis (MTCC 121), E. coli (UP 2566), Staphylococcus typhimurium (MTCC 98), Shigella dysenteraie, P. aeruginosa, P. vulgaris (ATCC 6380)*, while α-CBT-diol only inhibited the growth of *S. aureus, B. subtilis (MTCC 121),* and *P. vulgaris (ATCC 6380)* and was weaker than β-CBT-diol*.* β-CBT-diol also demonstrated broad-spectrum antifungal activity, inhibiting the tested fungi including *Candida albicans, Fusarium chlamydosporum, Rhizoctonia bataticola, Aspergillus niger,* and *Alternaria alternata*, while α-CBT-diol had a weaker inhibition on *C. albicans, F. chlamydosporum, A. niger,* and *A. alternata.* These findings highlight β-CBT-diol’s potential as promising antimicrobial scaffolds.

Some reports showed that β-CBT-diol attenuated HIV neurotoxicity by reducing glutamate release independently of viral replication and inflammation. Furthermore, β-CBT-diol could be used to resist HIV virus replication, HIV-associated neurocognitive disorders and HIV virus-induced inflammation (Andrew Ferchmin et al., 2012; Yan et al., 2019b). Due to the lack of more detailed information, the application of HIV and HIV- related diseases needs to be carefully verified.

## 5 Tobacco extracts

**FIGURE 4:**
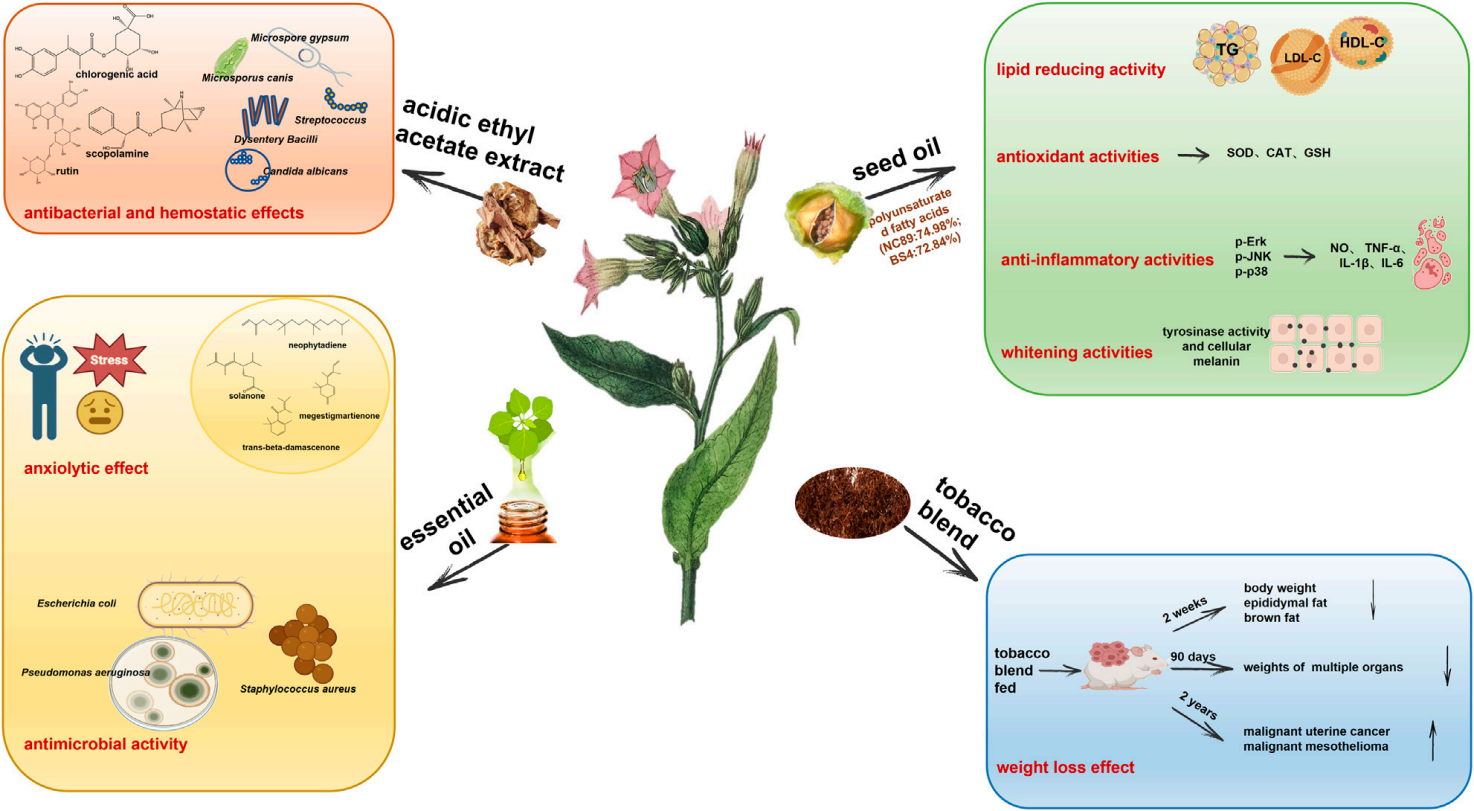
Tobacco plant extracts including acidic ethyl acetate extract, seed oil, essential oil, and tobacco blend have biological activities in antibacterial and hemostatic effects, anxiolytic effect, antimicrobial activity, lipid reducing activity, antioxidant activities, anti-inflammatory activities, whitening activities, and weight loss effect.

There are few studies in modern pharmacology on tobacco plant extracts, and most focus on the extraction and purification of the active substances such as nicotine and solanesol. In fact, apart from inhaled tobacco smoke, the early external use of tobacco leaves and their juice in disease treatment has been the inspiration for the application of tobacco extracts in modern medicine in anti-inflammatory, analgesic, bacteriostatic and hemostatic applications. For example, chewing green tobacco leaves in your mouth can help with oral mucositis. A heated mixture of tobacco and salt was crushed and applied to the root of the cervical gland to treat bacteriostasis, hemostasis and inflammation. Tobacco powder can also be applied to locally cure cuts or burns. The external application of tobacco can also treat the bites of toxic reptiles and insects, skin molds and ulcers (Charlton, 2004; Sanchez-Ramos, 2020). The medical significance of tobacco extract in modern medical research is mainly manifested in the continuation and in-depth proof of early records, as demonstrated in the following sections (Figure 4).

### 5.1 Antibacterial and hemostatic effects of tobacco acidic ethyl acetate extract

The acidic ethyl acetate extract of tobacco has strong broad-spectrum antibacterial and rapid hemostatic effects (Chen, 2017). *In vitro* bacteriostasis experiments based on the filter paper method, 96 well plate micro broth dilution method and minimum bactericidal concentration determination show that acidic ethyl acetate tobacco extracts have inhibitory effects, in the order from strong to weak, on *Dysentery Bacilli*, *Streptococcus*, *C. albicans*, *Microsporus canis*, *Trichophyton rubrum*, *Microspore gypsum* and *A. niger*, with the minimum inhibitory concentration and minimum bactericidal concentration of 1.56 mg/mL and 3.12 mg/mL, respectively. After the extract was intragastrically administered to mice for 1 week, the coagulation time of mice was observed after tail cutting. The coagulation time of the mice in the acidic ethyl acetate extract (0.4007 g/kg) group was about 154 s, which was 37.4% shorter than that of the blank control group, and close to that of the Yunnan Baiyao positive group. Its effective constituents include β-sitosterol, 3-O-β-D-glucopyranosyl-stigmasterol, anisodamine, quercetin, 7-hydroxy-6,6′-dimethoxy-3,7′-O-bis-coumarin, gallic acid, caffeic acid, chlorogenic acid, kaempferol 3-O-rutinoside, p-hydroxybenzoic acid and rutin after analysis by NMR and mass spectrometry. Of these constituents, chlorogenic acid, scopolamine and rutin may play a major role.

### 5.2 Tobacco seed oil

#### 5.2.1 Lipid reducing activity of tobacco seed oil

Tobacco seed oil is an excellent vegetable oil resource. The average oil content of tobacco seeds is about 39.4% and the content of unsaturated fatty acids is high (87.8%–89.4%), which is 7.5–8.5 times higher than that of saturated fatty acids. The content of linoleic acid is high (71.6%–75.36%), and its fatty acid composition is close to that of grape seed oil. The content of essential amino acids is higher than that of olive oil (Su, 2016; Yang et al., 2019). Su et al. (Su, 2016) systematically studied the toxicology and lipid-lowering activity of tobacco seed oil and found that the LD50 of tobacco oil for mice was more than 21,500 mg/kg, and no genotoxicity was found in a *Salmonella Typhimurium* test, bone marrow cell micronucleus test or chromosome aberration test. When tobacco oil (5, 10 and 20 g/kg) is added to the feed, there is no observed adverse effect on rats in the long-term feeding experiment (30 days), suggesting that tobacco oil is a vegetable oil without acute or chronic toxicity or mutagenicity to animals. Further research has been conducted on hyperlipidemia in Sprague-Dawley male rats models. The intragastric administration of tobacco seed oil (2, 4 and 6 mL/kg per day) has the ability to reduce triglycerides, low-density lipoprotein cholesterol and increase high-density lipoprotein cholesterol in serum, as well as decrease atherosclerosis index and ameliorate steatosis of liver cells. Hence, tobacco seed oil is expected to become an oil product with health benefits, especially for patients with hyperlipidemia, or cardiovascular disease.

#### 5.2.2 Antioxidant, anti-inflammatory, and whitening activities of tobacco seed oil

Seed oil of tobacco strain NC89 and BS4 are rich in polyunsaturated fatty acids (NC89: 74.98%; BS4:72.84%) that much higher than the values reported for other important food oils such as olive oils (25%), soybean oils (50.59%), and sesame oils (46%). Linoleic acid is the most abundant polyunsaturated fatty acid in NC89 and BS4. These two kinds of oil exerted the 1) antioxidant activity by scavenging ABTS, OH-, O2- radical, inhibiting ROS accumulation and enhancing SOD, CAT activities and GSH in H_2_O_2_-induced HepG2 cells 2) anti-inflammatory activity by inhibiting the expressions of NO, TNF-α, IL-1β, and IL-6 in LPS-induced RAW.264.7 cells through down-regulating the p-ERK, p-JNK, p-p38, and 3) whitening activity by inhibiting tyrosinase activity and cellular melanin production in melanoma B16 cells (Gu et al., 2022). These indicate that tobacco seed oil is a valuable and advantageous oil resource in food and cosmetic applications.

### 5.3 Weight loss effect of tobacco blend

Tobacco blend feeding can alleviate obesity and obesity-linked metabolic disorder in mice. When 27.46 g/kg of tobacco blend was added to the basic diet, after 2 weeks of feeding, the weight of mice was decreased by 16.3%, and the weight of epididymal fat and brown fat decreased by 67.6% and 42%, respectively, without inflammation. The increase in physical activity and the decrease of respiratory exchange rate in mice suggest that tobacco feeding can induce triglyceride lipolysis of adipose tissue to provide fatty acids. Without changing insulin sensitivity, the insulin level in plasma increases 3.6-fold and the blood sugar content decreases (Liu et al., 2018). Theophilus *et al.* (Theophilus et al., 2012; Theophilus et al., 2015) carried out feeding experiments lasting for 90 days and 2 years, with feeds including basic diet, tobacco blend mixed feed, tobacco blend water extract mixed feed and nicotine. Results indicate that plasma nicotine concentration in the middle and low dose groups is about 10–37 ng/mL, and that in the high dose group reaches 89 ng/mL, which is close to that in the plasma of smokers (10–50 ng/mL). The long-term tracking results show that the feed utilization rate of rats and mice is also decreased and, compared with obese mice, their weight is also significantly reduced (13%–28%), with decreased weights of organs such as brain, testis, salivary glands, adrenal glands, epididymis, pituitary gland and liver. After clinical observation, ophthalmological examination, toxicity dynamic analysis, clinical pathology, gross pathology and histopathological analysis, there was no increase in toxicity or carcinogenicity. During the 2-year feeding process, in addition to spontaneous and concomitant diseases, the incidence of malignant uterine cancer in female mice and malignant mesothelioma of the epididymis in male mice increases significantly. However, a decreasing tendency was observed in benign breast adenoma (female mice fed with tobacco), malignant skin basal cell carcinoma (female mice fed with water extract of tobacco blend), and benign thyroid follicular cell adenoma (male rats fed with water extract of tobacco blend). The incidence of tumors is also related to the genetic background and age of the mice, which require additional investigation.

Regarding tobacco plant extracts, on the premise of avoiding the toxic and carcinogenic substances ingested by smoking, the abundant medicinal substances in tobacco could exert safe and effective function in anti-inflammation, anti-bacteria, and fat and weight reduction. In the future, this smokeless tobacco product has the potential to play an important role in human healthcare and could also serve as a viable strategy to quit smoking.

### 5.4 Tobacco essential oils

As an important aromatic plant, tobacco contains more than 3,000 compounds and much more than other natural products (Mookherjee and Wilson, 1990). Tobacco essential oils may be the constituents that responsible for treating mental health problems such as anxiety and depression in the clinic (Berlowitz et al., 2020). Recent research has also found that it has antimicrobial effects (Stojanovic et al., 2000; Palic et al., 2002; Popova et al., 2015).

#### 5.4.1 Anxiolytic effect

Anxiety disorder is a prevalent and highly disabling mental health condition; however, there is still a lack of desirable therapeutic outcome, affecting up to 6% of the population during their lifetime (Maron and Nutt, 2017). According to the first report, the unique aroma of tobacco essential oils mainly contained neophytadiene and solanone, followed by megastigmatrienone and trans-beta-damascenone, which have an anxiolytic effect. Both Yunnan tobacco essential oil and Zimbabwe tobacco essential oil on male ICR mice in the light-dark box test and the maze test via inhalation and transdermal administration showed the anxiolytic effect through improving behavior and salivary corticosterone levels. Moreover, oral toxicity evaluation demonstrated that the concentrations of these oils were considered safe (Xie et al., 2021). However, we should carefully analyze the composition of essential oils to ensure that no toxic constituents are present, especially excessive nicotine residues which may have potential harmful effects. Purifying the main constituent for the future medical applications will be the most basic requirement.

Neophytadiene is the most noteworthy active metabolite in tobacco essential oil. In addition to tobacco, neophytadiene is also a major constituent of many plant essential oils, including *Jatropha curcas L (*Euphorbiaceae*), Acalypha segetalis, Zea mays* (Konstantopoulou et al., 2004; Aboaba et al., 2010; Adeosun et al., 2017). The anxiolytic-like, antidepressant-like, anticonvulsant, sedative effects and the anti-inflammation of neophytadiene (Bhardwaj et al., 2020; Gonzalez-Rivera et al., 2023) in the neuropharmacological actions may be the key point for tobacco essential oil, which has the effect not only on anxiolytic effect but also on the other mental diseases.

#### 5.4.2 Antimicrobial activity

Early reports showed that tobacco essential oil mainly containing neophytadiene and solanone in the middle leaves of Otlja and Prilep showed greater activity against the microorganisms *E. coli*, *S. aureus* and *P. aeruginosa* than that in the upper leaves or than the CO2 extracts (Stojanovic et al., 2000; Palic et al., 2002).

## 6 Conclusion

**FIGURE 5:**
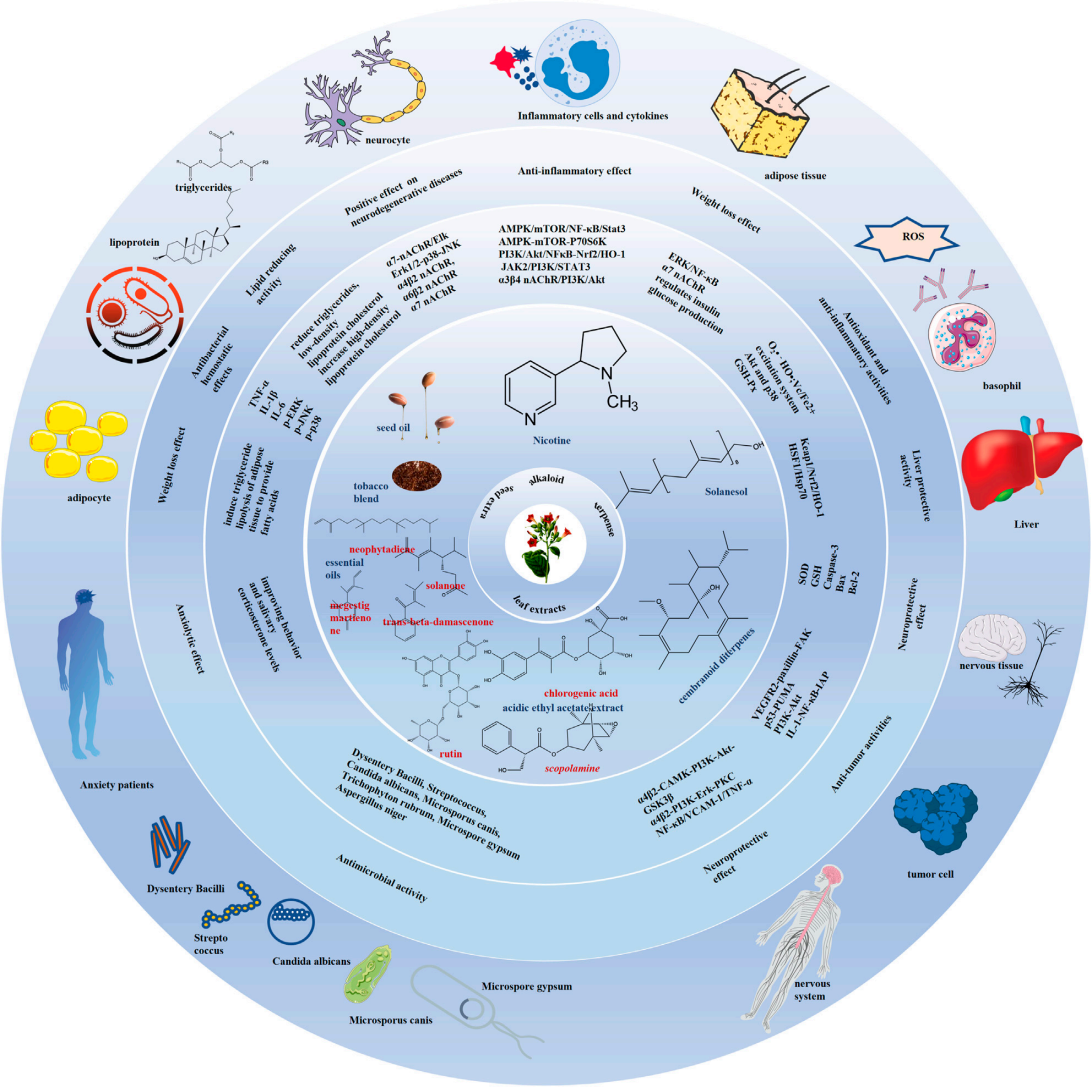
Summary of medical and pharmaceutical value of tobacco plant and its functional components. The tobacco and its components including nicotine, solanesol, seed oil, leaf extracts have neuroprotective, anti-inflammatory diseases and metabolic syndrome regulation effects through anti-oxidation, anti-lipid production, pro-lipid oxidation, pro-insulin sensitivity, anti-inflammation, anti-apoptosis and antibacterial activities. The main pathways of AMPK/mTOR/NF-κB/Stat3, PI3K/Akt/NFκB-Nrf2/HO-1, α4β2, α6β2, α7 nAChR/AKT/PI3K, α7 nAChR/ERK/NF-κB/iκB, Akt-p38/Nrf2/HO-1 and target proteins including the inflammatory factors IL-6, TNF-α, IL-1β, IL17, IFN-γ, IL-8, IL-4, IL-5, IL-1, IL-10, and PGE2, and the apoptosis markers bax, caspase-3, Bcl-2, and PARP are involved in their roles in regulating diseases.

Worth noting, this study is not intended to promote commercial tobacco smoking because it will never change the fact that tobacco smoke and its combustion products possess tobacco-specific carcinogens, which induce several malignancies. What we really advocate is that recognizing fresh tobacco bioactive metabolites is adding pharmaceutical value to this economically significant agricultural crop. The development of modern medicine and pharmacology, advances in compound separation, extraction and identification technology, and cross-disciplinary communication have been in step with research into and changing attitudes towards the tobacco plants. Effective functional metabolites of tobacco including nicotine, solanesol, seed oil containing high unsaturated fatty acids and linoleic acid, and leaf extracts mainly containing chlorogenic acid, kaempferol 3-O-rutinoside, p-hydroxybenzoic acid and rutin have emerged in many disease fields, especially in some difficult diseases: cranial nerve diseases including Alzheimer’s disease, Parkinson’s disease, Huntington’s disease, intracerebral and ventricular hemorrhage, autism, and bipolar disorder; inflammatory diseases including rheumatoid arthritis, ulcerative colitis, septicemia, multiple sclerosis, myocarditis, and periodontitis; antibacterial activity including the elimination of *E. coli, M. phlei, P. aeruginosa, S. aureus, Dysentery Bacilli, Streptococcus, C. albicans, M. canis, T. rubrum, M. gypsum, and A. niger*. They exert the function of anti-oxidation, anti-lipid production, pro-lipid oxidation, pro-insulin sensitivity, anti-inflammation, anti-apoptosis and antibacterial activities by regulating the main pathways of AMPK/mTOR/NF-κB/Stat3, PI3K/Akt/NFκB-Nrf2/HO-1, α4β2, α6β2, α7 nAChR/AKT/PI3K, α7 nAChR/ERK/NF-κB/iκB, Akt-p38/Nrf2/HO-1 and target proteins of inflammatory factors IL-6, TNF-α, IL-1β, IL17, IFN-γ, IL-8, IL-4, IL-5, IL-1, IL-10, PGE2 (Figure 5). Thus, tobacco has become a medicine and treatment choice to improve quality of life, and even prolong life span.

The tobacco plant should be a good resource for drug development. However, biomedical research data on the efficacy of tobacco is limited, probably due to the following reasons. At present, the development and use of tobacco are mainly focused on the cigarette industry. Government funding and support are insufficient for scientific research institutions working in this new field. Furthermore, public attitudes towards tobacco are still dictated by the harmful effects of smoking. Acceptance of the potential role of the tobacco plant in healthcare will require positive publicity. Considering the current development direction of the cigarette industry, tobacco is not attractive to talents in the biomedical and multi-disciplinary fields, and the vitality of scientific and technological innovation is relatively low. In this emerging field, reports on the structural identification of bioactive tobacco plant metabolites, and efficacy and safety studies, are scarce. Furthermore, extract separation and purification are challenging, resulting in a high cost for highly pure products. Pharmacological studies are mostly conducted on a limited number of metabolites, such as nicotine and solanesol, and specific diseases. Insufficient investigation on molecular mechanisms also leads to the termination of clinical experiments due to lack of specificity.

In the future, research should focus on drug analysis, separation and identification to build a molecular library of drugs from tobacco plant resources. Purification technologies and the purification output of pharmaceutical compounds should be improved and refined to lay a solid foundation for drug research and development. Moreover, the molecular mechanisms should be studied in more depth to thoroughly understand the pharmacological and toxicological mechanisms and pharmacokinetics trait of tobacco and its metabolites so that the tobacco plant can play a positive therapeutic role in the treatment of human diseases.

## Figures and Tables

**Table:** 

**AMPK**	AMP-activated protein kinase
**Th2**	helper T cell 2
**mTOR**	mammalian target of rapamycin
**GATA3**	GATA binding protein 3
**NF-κB**	nuclear factor kappa B
**RORγτ**	RAR-related orphan receptor gamma
**Stat3**	signal transducers and activators of transcription 3
**SOCS3**	suppressor of cytokine signaling 3
**PI3K**	phosphoinositide 3-kinase
**MMP9**	matrix metallopeptidase 9
**Akt**	protein kinase B
**VEGF**	vascular endothelial growth factor
**Nrf2**	nuclear factor erythroid 2–related factor 2
**CRP**	C-reactive protein
**HO-1**	heme oxygenase 1
**HMGB1**	high mobility group protein B
**nAChR**	nicotinic acetylcholine receptor
**PAI-1**	plasminogen activator inhibitor-1
**ERK**	extracellular signal-regulated kinase
**ROS**	reactive oxygen species
**iκB**	ikappaB kinase
**AST**	glutamic oxalacetic transaminase
**IL-6**	interleukin 6
**ALT**	glutamate pyruvate transaminase
**TNF-α**	tumor necrosis factor-alpha
**IL-1β**	interleukin 1β
**LPL**	lipoprotein lipase
**IL17**	interleukin 17
**PPARγ**	peroxisome proliferator activated receptor gamma
**IFN-γ**	interferon gamma
**MCP-1**	monocyte chemoattractant protein-1
**IL-8**	interleukin 8
**Bax**	BCL2 associated X apoptosis regulator
**IL-4**	interleukin 4
**LPS**	lipopolysaccharide
**IL-5**	interleukin 5
**SOD**	superoxide dismutase
**IL-1**	interleukin 1
**GSH-Px**	glutathione peroxidase
**IL-10**	interleukin 10
**MDA**	malondialdehyde
**PGE2**	prostaglandin E2
**Vc**	vitamin C
**JNK**	c-Jun N-terminal kinase
**GSH**	glutathione
**SIRT6**	Sirtuin 6
**LDH**	lactate dehydrogenase
**FGF2**	fibroblast growth factor 2
**SIRT1**	Sirtuin 1
**P70S6K**	ribosomal protein S6 kinase beta-1
**Bcl-2**	B-cell lymphoma 2
**MAdCAM-1**	mucosal vascular addressin cell adhesion molecule 1
**FAK**	focal adhesion kinase
**ZO-1**	zonula occluden-1
**ATP**	adenosine triphosphate
**Th1**	helper T cell 1
**p-gp**	glycoprotein
